# Errors and Action Monitoring: *Errare Humanum Est Sed Corrigere Possibile*

**DOI:** 10.3389/fnhum.2019.00453

**Published:** 2020-01-09

**Authors:** Franck Vidal, Boris Burle, Thierry Hasbroucq

**Affiliations:** Aix-Marseille Université, CNRS, LNC UMR 7291, Marseille, France

**Keywords:** action monitoring, errors of action, error correction, post-error slowing, partial errors, error negativity

## Abstract

It was recognized long ago by Seneca through his famous “*errare humanum est*.” that the human information processing system is intrinsically fallible. What is newer is the fact that, at least in sensorimotor information processing realized under time pressure, errors are largely dealt with by several (psycho)physiological-specific mechanisms: prevention, detection, inhibition, correction, and, if these mechanisms finally fail, strategic behavioral adjustments following errors. In this article, we review several datasets from laboratory experiments, showing that the human information processing system is well equipped not only to detect and correct errors when they occur but also to detect, inhibit, and correct them even before they fully develop. We argue that these (psycho)physiological mechanisms are important to consider when the brain works in everyday settings in order to render work systems more resilient to human errors and, thus, safer.

## Introduction

On Saturday, August 3rd, 1985, two trains frontally collided on the same unique railway line portion between the Flaujac and Assier train stations in France. Thirty-five people died in this catastrophe, henceforth termed the “Flaujac accident,” and many others were severely injured. Two days later, on August 5th, the French newspaper “Le Parisien Libéré (1985)” headlined: “The awful error of one single man^1^.”

How could this major accident be possible? First, there were some structural reasons: only one railway line was available for both directions of circulation and, as a consequence, only one train at a time was supposed to use this unique line. Both trains had to pass each other at Assier train station, where two railway lines were available. Second, there were some circumstantial reasons: one train was late while the other one was waiting for departure in the other direction at Assier. The traffic controller gave the go signal to the train driver at the scheduled time of departure even though the other train (being late) had not reached the station yet. Third, although the traffic controller realized his error almost immediately, it was useless as there was no means of communication between the controller and the train driver.

Considering that a human error did occur, one can point to human error to explain the catastrophe, as did “*Le Parisien*.” However, explaining this tragedy by “the awful error of one single man” only, is quite short-sighted. Reason (2000) presented two classes of approaches in error management. The traditional one is the “person approach,” which focuses on the erroneous actions and considers them as abnormal or blameworthy behavior that should be prevented by writing new procedures, further developing disciplinary measures, threatening, shaming, re-training, and so on. A more recent so-called “system approach” consists of recognizing that “… humans are fallible, and errors are to be expected… having their origins not so much in the perversity of human nature as in ‘upstream’ systemic factors.” (Reason, 2000, p. 708). If we consider the case of the Flaujac accident, although the traffic controller did realize his error almost immediately, once he had carried out the wrong action, the following events necessarily developed out of his control. Suppose that (as it has been the case later) he could have contacted the train driver by radio, this major accident would have been avoided.

This tragedy shows that the error was detected, as is the case for a large majority of human errors realized, such as those of human operators involved in integrated complex tasks (Amalberti, 2013); if radio communication had been allowed, it would have been corrected, thus avoiding this catastrophe. We show in this review that the same holds true for more elementary behaviors, such as those involved in sensorimotor information processing realized under times pressure: (1) the human information processing system is equipped with an action monitoring or supervisory system, which allows very fast detection and correction of inappropriate behaviors. Allowing these fast corrections might improve the safety of man–machine systems; (2) this action-monitoring system also acts to prevent errors (when possible), to detect abnormalities, correct errors at their very beginning (before they manifest or just after they are committed), and, finally, to modify response strategies for the future when, sometimes, this action monitoring has failed in its prevention and correction functions; certain conditions hamper the proper functioning of the action monitoring system. Identifying them can help avoiding deleterious situations and, therefore, contribute to reduce the number of human errors; (3) it should be clear that errors are integrated in the normal architecture of human information processing, which may contribute to lending support to the premises of the system approach; and (4) some physiological indices of action monitoring can be used for improving man-machine interactions.

More than 50 years ago, Rabbitt P. M. (1966), in his pioneering work on errors in sensorimotor information processing realized under time pressure, stated that “The speed with which errors are recognized and corrected is an important consideration in most industrial tasks.” (p. 264). We do not pretend here to survey the whole scientific literature for all forms of possible errors. We will mainly focus on errors occurring in sensorimotor information processing realized under time pressure, where a neuroscientific approach allowed for the revealing of hidden mechanisms that were quite difficult (or almost impossible) to evidence on the basis of behavioral analysis alone.

Even in this case, a taxonomy of errors is necessary. One can broadly distinguish between two types errors: perceptual failures and motor errors (e.g., Navarro-Cebrian et al., 2013). Perceptual failures can have two different origins: first, they can have their origin in the nature of the stimuli (poor quality, noised, hard to discriminate one from another, etc.) and, therefore, these failures cannot be considered as failures of the information processing system; second they can also be rooted in inappropriate perceptual operations (e.g., erroneous perceptual decision). On the contrary, errors of action purely reveal a failure of information processing operations (e.g., erroneous response selection processes, erroneous response execution, etc.). We will therefore concentrate on errors of action. A primary question of importance is concerned with how we react to errors, measured as post-error behavior. One of the first articles of Rabbitt and his colleagues’ pioneering work on this topic was entitled “What does a man do after he makes an error?” (Rabbitt and Rodgers, 1977, p. 727). We begin this review by examining this point.

## What Does a Man/Woman Do After He/She Makes an Error?

**Figure 1:**
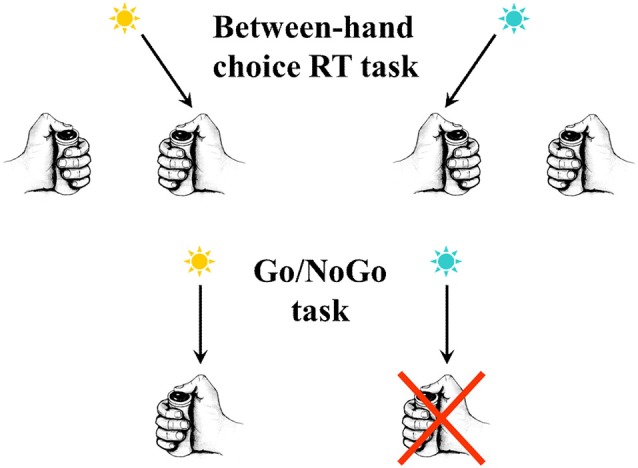
In the upper part of the figure an example is schematized of a between-hand choice reaction time (RT) task. In the present example, according to the color of the response signal, subjects must press a button as soon as possible after its presentation, using the right thumb if the signal is blue and the left thumb if the signal is yellow. In the lower part of the figure an example is schematized of a Go/NoGo task. Only one response is possible (here a button press with the right thumb). Nevertheless, a decision has to be taken. In the present example, according to the color of the signal, subjects have to press the button as soon as possible after its presentation if it is yellow, but they do not respond if it is blue.

When subjects have to make fast decisions under time pressure, they occasionally commit errors. In sensorimotor activities, the prototypical situation for studying fast decisions under time pressure corresponds to the reaction time (RT) paradigm, and much has been learnt about action monitoring from RT tasks in human (and non-human) subjects (see Figure 1 for an illustration of a RT task). By studying “what does a man do after he makes an error,” in RT tasks, it has been recognized from the very beginning that an action monitoring system is at work, in parallel with information processing operations. First, it was observed that, when required, subjects performing a choice RT task could correct most of their errors once they had been committed, even in absence of any external error signal (Rabbitt P. M. A., 1966; Rabbitt, 2002); moreover, mean correction time (i.e., the delay separating the error from the correcting response) was much shorter than mean correct RTs (and even shorter than RTs of the types of trials leading to the shortest RTs, i.e., repetition of the stimulus and repetition of the response)^2^. This demonstrated that the subjects’ “…internal monitoring of their own responses allowed them to correct errors more quickly than they responded to any external signal from the display” (Rabbitt P. M. A., 1966, p. 438). It must therefore be admitted that subjects can rely upon a fast internal signaling of their errors. In other words, humans can very quickly correct their errors on the basis of internal signals. If errors are quickly corrected, one has to admit that they are detected earlier (before error correction), that is even more quickly. These observations have been reproduced several times after (e.g. Fiehler et al., 2005). Therefore, it must be concluded from these observations that humans are endowed with an action monitoring system that allows them correcting their errors after they have been detected at the very moment when these errors were committed.

The effects of the action monitoring system are not confined to the ongoing trial but also concern upcoming trials: RTs of trials following an error are longer than RTs following correct trials (Rabbitt P. M., 1966; Rabbitt, 1969; Laming, 1979; Smith and Brewer, 1995; Allain et al., 2009). This effect, called post-error slowing (PES), may go beyond the trial immediately following (N+1) and may persist (although smaller) during N+2 trials (e.g., Rabbitt, 1969; Burns, 1971; Laming, 1979; Forster and Cho, 2014) or beyond (Forster and Cho, 2014).

A straightforward explanation of PES would be that subjects process information more carefully before giving their response on the trial(s) following an error, which would lengthen RTs (e.g., Laming, 1979; Dutilh et al., 2012). This seems quite reasonable if one also considers that, in these errors of action, RTs of errors are generally shorter than RTs of correct trials, suggesting that errors occurred when information processing has been superficial or incomplete because not long enough. This interpretation was supported by the fact that PES has been found to correlate with post-error accuracy (Hajcak et al., 2003; Forster and Cho, 2014; but see Danielmeier and Ullsperger, 2011; Pereiro et al., 2018).

However, as noted by Burns (1971) and, more recently, emphasized by Notebaert et al. (2009), the functional interpretation of PES as a simple reflection of more careful behavior after an error is not that straightforward.

First, the magnitude of PES depends on the duration of the interval separating the erroneous response from the stimulus of the following (N+1) trial (Rabbitt, 1969; Burns, 1971; Jentzsch and Dudschig, 2009). Second, post-error accuracy may not necessarily increase after an error (and it sometimes decreases), even when PES is present (e.g., Rabbitt and Rodgers, 1977; Fiehler et al., 2005; Hajcak and Simons, 2008; Carp and Compton, 2009; Notebaert et al., 2009; Valadez and Simons, 2018; Parmentier et al., 2019). This latter point is of importance given that, if PES is to be interpreted as an effect of more cautious behavior, one should expect that, subjects being more cautious, the likelihood of committing another error is reduced. However, as stated above, post-error improvement in accuracy (PEIA) is not always present and, in some instances, as indicated above, accuracy is even reduced.

Burns (1971), proposed that an orienting response (Sokolov, 1963a) to error commission is associated to response inhibition (as proposed by Sokolov, 1963b for any orienting response), which would slow down any subsequent action. This interpretation was put forward more recently by Notebaert et al. (2009), who provided convincing evidence for such an interpretation of PES. However, given that the orienting response is supposed to vanish quite quickly over time, this explanation cannot account for PES at the N+2 trial (and sometimes beyond) when evidenced (Burns, 1971; Forster and Cho, 2014). Moreover, several authors did report PEIA associated with PES (e.g., Laming, 1979; Marco-Pallarés et al., 2008; Danielmeier and Ullsperger, 2011; Seifert et al., 2011; Grützmann et al., 2014; Ruitenberg et al., 2014; Ceccarini and Castiello, 2018; Fischer et al., 2018; Pereiro et al., 2018; Overbye et al., 2019). This suggests that the orienting account of PES is only a part of this complex effect.

Given that: (1) PES decreases with the duration of the delay separating the error commission from the presentation of the stimulus of the next trial; and (2) that it may persist during an N+2 trial, Burns hypothesized that PES may have a double origin: a transient inhibitory orienting response followed by a response criterion adjustment towards a more cautious behavior. First, the orienting response might lead to error detection and, later, error detection would induce a more cautious behavior (see Wessel, 2018, for a detailed elaboration of this idea). In addition to this, several reports of PEIA associated with PES, Jentzsch and Dudschig (2009) provided further empirical evidence in favor of this idea. They reproduced (within and between subjects) the influence, on PES, of the duration of the delay separating the erroneous response from the stimulus of the following trial, PES being shorter (but still present) at long delays. Moreover, post-error accuracy was decreased at short delays, but it tended to increase at long delays. Even more importantly, the authors manipulated the perceptual difficulty of the task (low or high contrast). In accordance with the orienting account, contrast had no effect on post-error RTs (while it clearly appeared on correct ones). However, this absence of effect only held at short delays: at long delays, contrast affected post-error RTs, and the size of this effect was not different from that of correct trials. These latter observations were in contradiction with the orienting account of PES but fit with the response criterion account. In the same vein, although PES was not associated with PEIA, Valadez and Simons (2018) showed that longer post-error RTs were associated with more accurate responses.

Therefore, it seems that after an error, a transient orienting response exerts detrimental effects on subsequent information processing (if the delay separating the error from the next information to be processed is too short), which tends to increase the RT and error rate. Physiological evidence supports this view as the early response of the visual cortex is reduced after errors at short delays (Beatty et al., 2018). Later on, the early effects would vanish (e.g., the reduction of the cortical visual response disappears at long delays: Beatty et al., 2018) and a criterion adjustment towards more cautious behavior is set, which tends to increase RT while decreasing error rate. Depending on the delay separating the error from the next information to be processed, either the first or the second effect dominates (Burns, 1971; Danielmeier and Ullsperger, 2011; Wessel, 2018).

**Figure 2:**
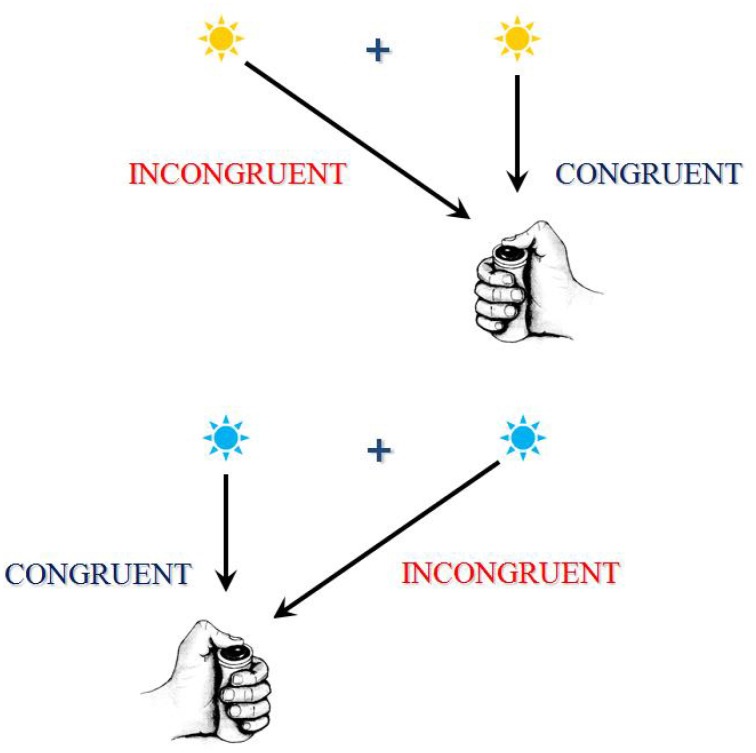
This figure exhibits an example of a conflict task. This task is a between-hand choice RT task. In the present example, according to the color of the response signal, subjects have to press a button as soon as possible after its presentation, using the right thumb if the signal is blue and using the left thumb if the signal is yellow. A central fixation point is presented on the screen, upon which the subject has to fixate his/her gaze. Now, the response signal can be presented on the same side as the required response (these associations are congruent) or on the opposite side (these associations are incongruent). Although the position of the stimulus is irrelevant to the task, RTs and error rates are higher on incongruent associations. The RTs and/or error rate differences between congruent and incongruent associations are called “interference effects” because, on incongruent associations, the contralateral response activated by instructions and anautomatic activation of the ipsilateral response are assumed to conflict. The resolution of this conflict takes time and sometimes fails, thus explaining the increase in RTs and error rates on incongruent associations. There are several other examples of conflict task; in these tasks, different mutually exclusive responses are assumed to conflict.

In certain choice RT tasks, often called “conflict tasks,” it is often assumed that different parts or different features of a same stimulus may, each, activate a specific response: one activation being be required by instructions, while another activation would be automatically triggered. When the different features of the stimulus concurrently activate two mutually exclusive responses, they compete and, in order to provide the correct response, this conflict must be solved. These situations are called “incongruent.” In other situations, called “congruent,” the different features of the stimulus concurrently activate the same response, and there is no conflict. Incongruent trials generate longer RTs and, in general, higher error rates than congruent trials. The difference between incongruent and congruent trials is called congruency or interference effect (see Figure 2 for an illustration).

After an error, provided that the delay between the response and the upcoming stimulus is long enough, the interference effect is reduced (Ridderinkhof, 2002; Ridderinkhof et al., 2002; de Bruijn et al., 2004; King et al., 2010; Fischer et al., 2018). Moreover, this effect is sensitive to the general structure of the tasks, being especially pronounced if congruent trials are more frequent than incongruent ones and absent if incongruent trials outnumber congruent ones (Ridderinkhof, 2002). This indicates that the action monitoring system is extremely versatile and can adapt to the broad context of the task.

To sum up, after making an error several things occur:

(1) Very fast corrections, if allowed, may occur, suggesting that subjects can rely upon an action monitoring system which very quickly generates internal error signals on the basis of internal information.
(2) RTs are slower and slowing is explained by (at least) two phenomena: (a) an early transient orienting response that hampers the information processing of the next response signal and increases RTs as well as error likelihood; and (b) the orienting response is followed (if the delay separating the error from the next response signal is long enough) by cognitive control-triggered strategic adjustments set to avoid committing new errors, which increase RTs but decrease error likelihood.
(3) In conflict tasks, the interference effects are reduced.

It is worth mentioning that, although the discovery of post-error behaviors dates back from the 60s, current research is still very active in this domain and largely aimed, now, at identifying the brain structures that are targeted by post-error action monitoring processes (e.g., Danielmeier et al., 2015; Beatty et al., 2018; Ceccarini et al., 2019) and also at identifying the brain mechanisms (e.g., Purcell and Kiani, 2016; Schiffler et al., 2016; Perri et al., 2017), brain structures (e.g., Schiffler et al., 2016; Zhang et al., 2017; Fu et al., 2019), or neurochemical systems (e.g., Danielmeier et al., 2015; Sellaro et al., 2015) that are involved in or responsible for post-error behaviors.

## What Does a Human Do When He Makes an Error?

As mentioned above, when subjects are required to correct their errors as soon as possible after their commission, this correction is very fast, and most errors are corrected (e.g., Rabbitt P. M. A., 1966). For example, in Experiment 2 of Marco-Pallarés et al. (2008), mean correction time was 179 ms ± 41 ms (to be compared to 382 ms ± 22 ms for correct responses and 314 ms ± 22 ms for errors); given that, for very simple responses (e.g., a thumb key press), the time separating EMG onset from the mechanical response in a between-hand two-choice RT task was at least 70 ms on average (e.g., Vidal et al., 2011), this means that the delay separating the error commission from the (EMG) onset of its correction was about 110 ms on average, with some corrections occurring even faster. This suggests that the action monitoring system was sensitive to errors extremely early—at the very moment when the error was committed or even before: “In some sense the subject knows that his erroneous response is wrong even before it is registered.” (Laming, 1979; p. 204). If this is the case, some physiological activities should distinguish between errors and correct responses at the very moment when the error is committed or even before the response is registered.

To examine the time course of physiological activities occurring when making an error (i.e., just before, during, or shortly after), it is mandatory to resort to high temporal resolution methods. Among those, electroencephalography (EEG) has provided much valuable information regarding action monitoring processes occurring just before and shortly after errors are committed.

**Figure 3:**
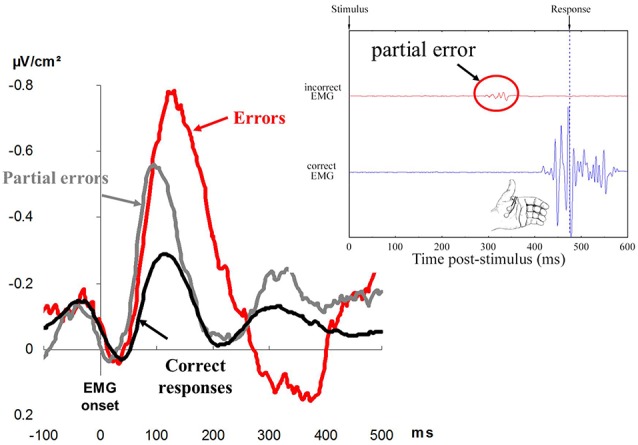
The left displays Error Negativies recorded on errors (red), partial errors (gray), and correct responses (black). Time is in abscissa in milliseconds, and amplitude of the surface Laplacian is in ordinate, expressed in microvolts per square centimeter (adapted from Vidal et al., 2000). The right displays a representative (real) correct trial containing a partial error. The black trace represents EMG recordings of the *flexor pollicis brevis* of the (correctly) responding thumb; the large EMG burst corresponds to production of the response. The red trace represents EMG recordings of the *flexor pollicis brevis* for the non-required response; the very small EMG burst (in the red circle) corresponds to the partial error. The amplitude of EMG is in ordinate. Time is in abscissa: a zero value for time corresponds to the presentation of the response signal, and the vertical dotted line corresponds to the moment when the contraction of the muscle is strong enough to trigger the mechanical response.

Falkenstein et al. (1991) examined response-locked event-related potentials (ERPs) on correct and erroneous responses. They discovered that a very large ERP, was evoked on errors, and this did not show up on correct responses. Since this ERP seemed to be specific to errors, it was named Error Negativity (Ne) or, later on, Error-Related Negativity (Gehring et al., 1993; see Figure 3 for an illustration). The Ne began before the mechanical response and culminated shortly after. This observation indicated that, even before the error was overtly committed, an action monitoring system detected the error or, at least, that something was going wrong.

When examining the Ne time locked to EMG onset instead of (mechanical) response onset, it appears that it began about 35 ms after EMG (e.g., Vidal et al., 2000) but clearly before the mechanical response. Therefore, the Ne is elicited during the execution of the error and begins before its overt commission. This is in line with the comment of Laming (1979) cited above; however, if Falkenstein et al.’s (1991) discovery demonstrated that something in the subject’s brain “… knows that his erroneous response is wrong even before it is registered.” (Laming, 1979), it is not warranted that the subject himself knows it at this very moment since the Ne can be elicited without error awareness. Moreover, the Ne is insensitive to such an awareness (Nieuwenhuis et al., 2001; Endrass et al., 2007; although it may not be the case for perceptual errors, e.g., Shalgi and Deouell, 2012).

On the basis of error correction behavior, Rabbitt P. M. A. (1966) demonstrated that the action monitoring system does not need to rely on external feedback to quickly correct errors. Now, one can wonder whether this system needs to rely on internal peripheral (sensory) feedback to detect malfunctions. A total of 35 ms (Ne onset) is, in principle, long enough to allow reafferent information to reach the somatosensory cortex (medial nerve electrical stimulation elicits a somatosensory response as early as 20 ms later). Allain et al. (2004b) showed that, in a totally deafferented patient (Cooke et al., 1985; Forget and Lamarre, 1987) who suffered a polyneuropathy affecting selectively the large myelinated sensory fibers, a large Ne was evoked on errors. Her action monitoring system could not rely on somatosensory feedback, and the early latency of the Ne could not be accounted for by auditory (e.g., button click) or visual (e.g., finger movement) evoked potentials. It can therefore be concluded, in line with the general opinion in the current literature, that the action monitoring system does not absolutely need peripheral (somatosensory, auditory, or visual) information to elicit the Ne and can rely on an internal simulation of the expected effects of the motor command on the basis of just central information.

Now, it has been shown that, provided that appropriate source separation methods are used (e.g., Laplacian transformation: Vidal et al., 2000; Independent Component Analysis: Roger et al., 2010), a smaller component systematically shows up on correct responses. This component on correct responses remained unnoticed at the beginning since it was hidden by large remote non-response-related components, present due to the volume conduction effects. Separation methods allowed for disentangling overlapping effects and unmasked the presence of a smaller negative wave on correct trials. Its time course and topography were similar to those of the Ne once these separation methods were applied, which suggests that this component and the Ne are of same nature. Moreover, both were influenced in the same way by explicit monitoring conditions: if subjects were explicitly asked to rate the accuracy of their responses, both the Ne and the post-response component elicited on correct trials were enhanced, as compared to a non-rating condition (Grützmann et al., 2014). Finally, the fact that intracerebral recordings in humans pointed to the same generator, in the supplementary motor area proper, for both components (Bonini et al., 2014) also supports the view that they are of the same nature. In other words, a large Ne is elicited on errors, whereas a smaller one is elicited on correct responses.

This indicates that the action monitoring system is always at work (even on correct trials), but its reaction is usually stronger on errors. These effects suggest that each response type, whether correct or erroneous, is evaluated on-line. Although the functional significance of the Ne is far from settled—and this question is out of the scope of the present article—this means that the brain is equipped with a system which function is to take into account response correctness at the very moment when it could occur (at least in sensorimotor activities when carried out under time pressure). Therefore, it is clear from what precedes that the functional architecture of the human brain is not organized so as to function in an errorless manner and that, as a consequence, there exists in the brain a system set to take this fact into account; this action monitoring system checks for possible dysfunctions, as indicated by the sensitivity of the Ne to correctness.

It has been demonstrated that the action monitoring system is not only competent for errors regarding the “what,” it is also competent for errors regarding “when.” Indeed, if a deadline is set in a choice RT task, correctly selected responses occurring after the deadline evoke a larger Ne than correct “on time” responses (Johnson et al., 1997; Luu et al., 2000; De Bruijn et al., 2005). Moreover, the later the response, the larger the Ne. Therefore, performance regarding “when” the response is issued may also be supervised by the action monitoring system, suggesting that this system might have access to an internal clock.

It noteworthy to indicate here that the Ne belongs to a general class of so-called “error potentials” related to the detection of abnormal functioning and action monitoring, regardless of whether this detection relies on internal information (e.g., the Ne) or on external information carried by a feedback. This feedback can be explicitly delivered by the experimenter (in this case the error potential is called feedback-related negativity: see Miltner et al., 1997; Walsh and Anderson, 2012 for a review), or it can be implicitly contained in the effects of the subject’s action from which feedback information is extracted and error potentials are evoked. These error potentials are assumed to represent an identical family of processes and to share, at least in part, common brain generators.

More peripheral observations also argue in favor of the existence of an action monitoring system acting during response execution but before error commission.

In a between-hand choice RT task involving a right or a left thumb keypress, Allain et al. (2004a) recorded surface EMG of the *flexor pollicis brevis*; they observed that the EMG burst corresponding to errors was smaller than that of correct responses. As a consequence, although the RT of errors was shorter than that of correct responses, the delay separating EMG onset from the mechanical response was longer on errors than on correct responses, as if a tentative response inhibition occurred on errors during their execution. One might argue that, on errors, even the execution processes were defective. However, the initial slope of the EMG burst was identical on correct responses and on errors, which suggests that the motor command was initially similar for both correct and erroneous responses (see Allain et al., 2004a, for a more detailed discussion on this point). The EMG bursts began to differ (on average) between correct responses and errors about 30 ms after EMG onset. These effects have been reproduced later (Meckler et al., 2011; Rochet et al., 2014; Roger et al., 2014). Once again, this means that shortly after EMG onset, but clearly before error execution, an action monitoring system triggered a tentative inhibition, maybe to avoid the error. It must be noted that: (1) brain activity indicating errors and erroneous responses began to differ about 20 ms earlier, as it takes about 20 ms for information to travel from the primary motor cortex to hands muscles; and (2) in order to trigger a corrective behavior, error detection must have occurred before. Therefore, it seems that before—or at least at the very beginning of—the execution of an error, the action monitoring system detected that something was going wrong.

To sum up, in sensorimotor activities under time pressure, at the very moment when humans are producing their response, several things happen:

(1) EMG recordings of the muscles agonists to the responses reveal an early tentative inhibition of the response, and this early within-trial inhibition implies that error detection has occurred even earlier;
(2) brain structures are responsive to correctness, whether decisional or temporal, and this sensitivity is revealed on EEG recordings by the early influence of response correctness on the amplitude of the Ne.

Both early response inhibition on errors and brain responsiveness to correctness indicate that, very early, the action monitoring system is able to detect on-line (i.e., within trial) that something is “going wrong.”

## What Does a Human Do When He Is About to Make an Error?

Although peripheral response inhibition seems to index a desperate act of control, if it always fails, one may wonder what its functional significance is. Other analyses, still based on EMG recording, indicate, however, that this inhibition often succeeds.

In between-hand choice RTs tasks, Eriksen et al. (1985) and Coles et al. (1985) observed that correct responses were sometimes preceded by a small sub-threshold EMG activation (insufficient to trigger an overt response) on the “wrong” side. These results have often been reproduced ever since (e.g., Masaki and Segalowitz, 2004; Allain et al., 2009; Maruo et al., 2017; Ficarella et al., 2019). Given that these activities occur in the “wrong” effector, they have been considered to be partial errors (see Figure 3 for an illustration), and “analysis of these partial errors reveals that things may be going wrong with the system, even when its final output, the overt behavioral response, is correct” (Coles et al., 1995, p. 130). This is very important from a human factor point of view since “things may be going wrong” only covertly, and there is no behavioral evidence for such processing failures. The positive side is, however, that these partial errors can be overcome; that is, they can be detected, inhibited, and corrected on time. This is suggested by the fact that partial errors share several properties with overt errors.

For example, sequential effects are extremely similar between errors and partial errors. Post-partial error slowing has also been evidenced (Allain et al., 2009), although it is much smaller than PES; however, it must be noticed that: (1) the delay separating partial error onset from the stimulus of the next trial is longer than that separating errors from the following stimulus and, as it has been indicated above, the longer this delay, the smaller PES; and (2) partial errors are separated from the next stimulus by the correct overt response, and it is noteworthy that Fiehler et al. (2005) failed to evidence any PES following corrected overt errors. More recently, Ficarella et al. (2019) added new insight regarding the nature of these sequential effects. Post (partial)-error slowing occurs in a subset of partial errors only—the minority (about one third: Rochet et al., 2014) of the partial errors which are consciously detected.

Moreover, in conflict tasks, a reduction of the interference effect has been reported after a trial containing a partial error (Burle et al., 2002b), as is also the case after a full-blown error (Ridderinkhof, 2002).

Error-related sequential effects are not confined to the trials following the errors, they can also be concerned with the preceding trials. Indeed, RTs of correct responses preceding an error are generally faster than RTs preceding another correct response (Smith and Brewer, 1995; Allain et al., 2009). The same effect has been observed before partial errors: RTs preceding partial errors were faster than RTs preceding a correct response (Allain et al., 2009). If we admit that PES may, in part, reflect more cautious behavior triggered by action monitoring, these effects suggest that before-errors and also partial-errors subjects have adopted an error-prone, less cautious behavior with lowered control.

Finally, partial errors usually elicit a Ne that is time locked to their onset. This Ne has an earlier latency (Masaki and Segalowitz, 2004; Maruo et al., 2017), and its amplitude is, in general, larger than that of correct responses but smaller than that of overt errors (e.g., Scheffers et al., 1996; Vidal et al., 2000; Roger et al., 2010; Maruo et al., 2017). In some instances, however, the amplitude of the Ne of partial errors may be as large as that of full-blown errors (Carbonnell and Falkenstein, 2006). Note that in this latter case, however, partial errors were defined not on the basis of subthreshold EMG activity but on the basis of subthreshold force production, which likely accounts for the results of Carbonnell and Falkenstein (2006).

The preceding observations suggest that partial errors were “small errors” that could be corrected on time or, even better, erroneous response activations that remained small because they were quickly detected, inhibited, and corrected on time.

Moreover, the larger the size of the partial error, the longer it takes to correct it (i.e., to emit the correct response once this partial error has been suppressed) and the larger the Ne (Burle et al., 2008); intracerebral recordings (LFPs) in humans allowed for the examining of the (co)relations between partial errors EMG bursts and the Ne on a trial-by-trial basis. Partial errors LFP activity peaked when partial errors were inhibited and stopped when the correction began (Bonini et al., 2014), suggesting that: (1) large partial errors, being closer to a full error, evoked larger Nes and required more processing before being corrected; and (2) that the Ne might represent an alarm signal at work until the correction is completed.

Sometimes, the action monitoring process allowing these fast inhibitions fails and a full-blown error occurs. However, a sign of this tentative inhibition still persists on errors: the EMG burst is smaller on errors than on correct responses (see preceding section).

If this interpretation is correct, partial errors reveal, once again, that information processing is not organized to function in an error-free manner; however, correction is possible and nearly always attempted. It is to be noted that the deafferented patient studied by Allain et al. (2004b) also presented partial errors that evoked a Ne whose amplitude was intermediate between that of the Ne evoked by correct responses and errors. Of course, these partial errors could hardly be detected (and corrected) on the basis of somatosensory information, and visual or auditory information were absent due to a partial error, failing to result in movement or a a much-needed button click.

Partial errors, when compared to errors, allow for the calculation of a correction ratio: the number of partial errors divided by the total number of incorrect activations (partial errors plus errors). This correction ratio (expressed between zero and one) can be considered as a measure of the efficiency of the supervisory system (Burle et al., 2002b).

If this opinion is correct, there may exist two (independent) factors contributing to variations of accuracy: those resulting from variations in the reliability of the information processing chain and those resulting from variations in the efficiency of the action monitoring system. Two datasets can exemplify the contribution of each of these factors.

van de Laar et al. (2012) showed that, in a choice RT task, 8 or 12-year-old children committed about three times more errors (9.7%) than 20-year-old adults (3.5%). The same effect showed up on partial errors (29.05% vs. 10.1%; calculated based on table 2 in van de Laar et al., 2012, p. 327). On this basis it is possible to estimate the correction ratio of the children (0.76) and young adults (0.74). It is clear that the increase in the error rate of children cannot be attributed to a failure of the action monitoring system as they could correct their errors at least as well as young adults. On the contrary, the differences in error rates are completely explained by a larger proportion of incorrect activations (partial errors plus errors).

The reverse is observed in Parkinsonian patients receiving Dopa therapy. Fluchère et al. (2015) analyzed errors, partial errors, and correction rates in Parkinsonian patients in two different conditions: off medication and on medication. They showed that error rates were increased by dopa therapy, and that this increase was completely explained by a decrease of correction rates—a decrease in the efficiency of the action monitoring system.

Finally, in healthy adults, it has been demonstrated that a large part of the increase in error rates when subjects trade accuracy for speed in sensorimotor tasks is due to a lower efficiency of the action monitoring system: from 93% under accurate conditions, the correction rate may drop to 71% under speeded conditions (Burle et al., 2014).

The data presented up to now indicate that the action monitoring system is competent to supervise errors of selection and temporal errors. Recent data indicate that it is also competent regarding execution errors.

Meckler et al. (2017) examined partial correct responses, i.e., subthreshold EMG activities that were insufficient in triggering the correct response but were followed by an overt correct response (Questienne et al., 2018). First, the EMG burst corresponding to the “corrective” response of the partial correct activation was larger and more phasic than that of the “corrective” response of partial errors or that of “pure” correct responses (“corrective” responses of partial errors and “pure” correct responses did not differ from one another). This suggests that the execution of the response force was insufficient, and that it was “overcorrected” when executing the “corrective” response. Second, the force exerted on correct trials preceding partial correct trials, although sufficient to trigger the mechanical response, was smaller than that of pure correct trials preceding other pure correct trials. This was not the case for trials preceding partial error trials. This suggests that before a partial correct trial, subjects were in a phase where the representation of the to-be-produced force was not very accurate^3^. Finally, the Ne evoked by partial correct responses was larger than that of pure correct responses and marginally smaller than that evoked by partial errors. Given that the required force never varied throughout the experiment, subjects could program this parameter and did not have to select it at each trial. These partial errors can therefore hardly be attributed to a failure of the selection process. On the contrary, they appear to result from failures of the execution process^4^. As a consequence, one can conclude that the competence of the supervisory system also extends to execution errors.

To sum up, partial response activations first indicate that the action monitoring system is able to “on-line” detect, inhibit, and correct most incorrect activations. In other words, the supervisory system can trigger remedial activities not only between but also within trials. Only when these action monitoring processes fail does an overt error occur. Examining the correction ratio can determine which factors hamper the proper functioning of the action monitoring system. Second, the properties of partial correct responses indicate that the supervisory system not only monitors response selection and temporal accuracy, but also monitors execution accuracy. Third, although tasks like those presented here define errors in an all or none manner, it is unlikely that the same holds true for the supervisory system, which seems to evaluate performance on a more continuous scale. This is suggested by the fact that there is an increases in the amplitude of the Ne from correct responses to partial errors and from partial errors to full blown errors; the larger the size of the partial error (i.e., the closer it is to a full blown error), the larger the Ne. It could therefore very well be that the way certain tasks are prescribed is not in accordance with the representation of correctness set by the human action monitoring system.

Finally, from a human factors point of view, these observations show that the error rate alone is a rather poor reflection of the effects of experimental conditions or working conditions on information processing and/or action monitoring processes. If one seeks to counteract the effect of work deleterious situations on sensorimotor performances one has to first identify on which system (information processing chain or action monitoring system or both) these deleterious situations have an effect before envisioning any countermeasure.

## What Does a Human Do When He Might Make an Error?

Certain RT situations are risky, others are not. For example, in a simple RT task, the risk of committing an error of decision is null (if there are no “catch” trials—rare trials in which the response signals requires refrain responding), and the only possibilities of inappropriate behavior are errors of execution (e.g., insufficient response force), anticipations, or late responses in the case of a deadline.

On the contrary, in choice RT tasks, depending on the paradigm and speed-accuracy strategy, the risk of committing errors is much higher and, if the stimuli are unambiguous, most errors are decisional (with a small percentage of execution errors).

We will see in the following section that the action monitoring system is able to take into account this risk and sets specific mechanisms to prevent them.

In a between-hand choice RT task, it has been demonstrated that spinal excitability (as assessed by the Hoffmann reflex) is stable during most of the RT period; however, in its very last part, sudden changes occur just before EMG onset. The excitability of spinal motoneurons controlling the responding hand muscles increases, while that of spinal motoneurons controlling non-responding hand muscles (or, in other words, those involved in the not to be produced response) decreases (Hasbroucq et al., 2000).

In the same kind of paradigm, Burle et al. (2002a) studied the time course of intracortical excitability of the primary motor cortices (M1) thanks to the duration of the silent period (SP) evoked by transcranial magnetic stimulation. The SP corresponds to a transient drop in EMG activity, which extends for a short time period, just after the motor potential evoked by the magnetic stimulation. Contrary to the motor evoked potential, the origin of the SP is purely intracortical (e.g., Roick et al., 1993; Schnitzler and Benecke, 1994), and, as such, it has been used as a probe of intracortical excitability: the shorter the duration of the SP, the higher M1 excitability, while the longer the duration of the SP, the lower M1 excitability. In a between-hand choice RT task, Burle et al. (2002a) showed that after a certain delay from the time of presentation of the stimulus, the duration of the SP progressively decreased when the stimulated site is contralateral to the responding hand (involved in the response), while, concurrently, the duration of the SP progressively increased when the stimulated site is ipsilateral to the responding hand (involved in the non-required response). This means that before EMG onset, M1 involved in the response was activated, while M1 involved in the not to be produced response was inhibited.

In the same type of task, Tandonnet et al. (2011) demonstrated that the Motor Evoked Potentials (MEP) generated in the muscles involved in the response by the magnetic transcranial stimulation of contralateral M1 increased just before EMG onset, while the MEP of ipsilateral M1 decreased just before EMG onset. This dataset perfectly bridges the gap between the observations of Hasbroucq et al. (2000) and those of Burle et al. (2002a).

Still, in a between-hand choice RT task, response-locked ERPs showed that, shortly before EMG onset, a transient negativity developed over contralateral M1, while a positive wave developed over ipsilateral M1 (Vidal et al., 2003; van de Laar et al., 2012; Amengual et al., 2014)^5^. This EEG negativity/positivity pattern is to be considered the EEG counterpart of the activation/inhibition pattern evidenced by Burle et al. (2002a; see Burle et al., 2004, for a detailed discussion of this point). Considering that EEG recordings (even Laplacian-transformed) are much easier to perform than H-reflex or TMS measures, this activation/inhibition pattern has been explored thanks to EEG recordings as detailed below.

Given that the topic of this article concerns errors, one can guess that the working hypothesis regarding this pattern is that, although activation relates to the build-up of the motor command, inhibition is set to prevent errors on the “wrong” side.

If this is the case, when only one hand is involved and, as a consequence, there is no risk of committing an error, no ipsilateral inhibition should be evidenced. This situation is seen in Go/NoGo tasks where, although a certain type of decision is to be taken (doing or not doing), this decision is not about executing one of two (or more) movements (see Figure 1 for an illustration). In such a situation, contralateral activation is, of course, present because the movement must be triggered, but inhibition is absent (Vidal et al., 2011). This is a necessary but insufficient observation in favor of the idea that ipsilateral inhibition would reveal errors prevention. To support this hypothesis more firmly, it is mandatory to explicitly manipulate the risk of errors.

This can easily be achieved by manipulating the probability of each response in a between-hand choice RT paradigm, either in a classical 0.5/0.5 probability distribution for each response or in a biased 0.8/0.2 probability distribution for each response. The lower the probability of a response, the more unexpected it is and the higher the error likelihood for this response. While contralateral activation is unaffected by the probability bias, ipsilateral inhibition is affected. The higher the error rate [under the influence of (un)expectancy], the stronger the ipsilateral inhibition (Meckler et al., 2010). Moreover, in the unexpected condition (0.2 probability for the required response), where the error rate was the highest, there was an inverse correlation between error rate and strength of ipsilateral inhibition. The subjects who showed the strongest ipsilateral inhibition were those who produced the lowest error rate (Meckler et al., 2010).

In light of these results, it is noteworthy that 8-year-old and 12-year-old children of van de Laar et al. (2012), who commit about three times more errors and partial errors than young adults, completely lacked ipsilateral inhibition^6^.

In the experiment of Meckler et al. (2010), biased or unbiased conditions were administered in separate blocks. It is therefore possible that the action monitoring system had set the relative strength of possible inhibitions of each side before each block. Could it be possible that the relative strength of each inhibition would be set at each trial after the response signal?

To answer this question, Burle et al. (2016) manipulated the probability of compatible and incompatible trials in a conflict task while keeping the probability of all other events (stimuli attributes and responses sides) equal. Subjects had to respond according to the color of a stimulus that could either spatially correspond to the response (compatible trials) or spatially correspond to the not to be produced response (incompatible trials). In blocks of frequent incompatible trials, the error rate was quite low, and the strength of inhibition was not different for compatible and incompatible trials. On the contrary, in blocks of frequent compatible trials, the error rate for incompatible trials was quite high and ipsilateral inhibition was stronger on (rare) incompatible than on (frequent) compatible trials.

It should be stressed that, in the compatible frequent blocks, neither the compatibility nor the nature of the required response could be predicted. This demonstrates that the strength of ipsilateral inhibition was to be adapted on each trial, after the presentation of the response signal. These results highlight the high versatility of the action monitoring system which, in parallel with sensorimotor information processing and under time pressure, has to very quickly evaluate the nature of the risk (i.e., the stimulus response association: compatible or incompatible), the nature of the response (i.e., right or left) to be inhibited, and the strength to be set for this inhibition.

To sum up, in sensorimotor activities realized under time pressure involving a choice between several overt actions, the action monitoring system sets preventive inhibition of the possible erroneous responses, the strength of this inhibition predicts (between subjects) the error rate, the strength of this inhibition is highly dependent on the context, and, considering that the strength of this context-dependent inhibition can be set within trial, it can be concluded that the supervisory system is extremely flexible.

## Relevance to Human Factors and the Brain at Work in Everyday Settings

### The System Approach Still Needs Scientific (and Extra-Scientific) Support in the Real World; Basic Knowledge on Action Monitoring May Contribute to This Support

The fact that different specific action monitoring mechanisms take errors into account at different stages of sensorimotor information processing suggests that, for this type of processing, errors are part of the normal functioning of the human brain and are not just occasional unavoidable and undesirable dysfunctions. Put differently, errors are not simply unavoidable failures; on the contrary, errors are integrated into the normal structure of the human information-processing architecture. As a consequence, it seems reasonable to admit that the systems to which human operators belong should be designed to live with human errors and to avoid (or minimize the likelihood) that the consequences of these errors generate system failures. In other words, knowledge on action monitoring issued from basic research strongly advocates in favor of the idea that the “system approach” is much preferable to the “person approach” to improve safety. One could feel that such basic research arguments are useless (or come too late) since most scientists do consider that humans cannot completely avoid committing errors. Nevertheless, several researchers in the safety domain also indicate that, in the analysis of failures and accidents, the person approach is still pervasive—probably because, in this domain, there is a “… gap between research and practice” (Underwood and Waterson, 2013, p. 155), as stated by Catino (2008), “Though favoured by the scientists, the organizational function logic approach is in real life usually beaten by the individual blame logic” (p. 53). In the same vein, Bitar et al. (2018), in a case study, reported that “… the proposed solution was to re-train the individuals to reduce the likelihood of error in the future …”. More generally, Leveson (2011a) considers that, among the misleading assumptions widely shared in the safety domain, one can find the following: “(1) Most accidents are caused by operator error; and (2) rewarding “correct” behavior and punishing “incorrect” behavior will eliminate or reduce accidents significantly” (p. 61). The same author also considers that “These assumptions underlie the common behavioral approach to occupational safety” (p. 61), and that, in case of systems failures, “…if there are operators in the system, they are most likely to be blamed.” (Leveson, 2011b; but see also Ivensky, 2017a,b for similar views). These attitudes still correspond to the “person approach” criticized by Reason (2000). All these remarks from safety specialists indicate that the ‘system approach’ still needs (scientific and extra scientific) support, as opposed to the “person approach.” The data set reviewed here on action monitoring is one element (among others) that lends support to the “system approach” and might contribute to reinforcing its position.

### A Word of Caution Regarding the Ecological Validity of the Results From Basic Research Laboratory Tasks

Before examining the relevance to human factors and possible applications of the data set reviewed here further, a word of caution is needed regarding the ecological validity of the results obtained in the type of laboratory tasks presented here so far. Most of them are RT tasks that were realized with simple and often arbitrary stimulus-to-response mapping instructions (but think of a car driver when the traffic light turns red); they were realized in controlled laboratory conditions in which, unless attention is studied, no other task or information interfered with the processes under study. In other words, these processes are studied in their optimal working conditions, and one must admit that these optimal conditions are scarcely met in the real world. From these tasks, therefore, it is clearly not possible to directly infer what *should* be done in real world conditions to improve working environments for safety. Nonetheless, these tasks, because simple and controlled, allow for the proposing of some ideas about what *should not* be done in these real-world situations; if limitations of a given process can be found under optimal conditions, these limitations are likely still present in sub-optimal ones (might even be worse) and can reasonably be taken into account for applications when the brain works in everyday settings.

For example, in conflict tasks, under optimal laboratory conditions, incongruent stimulus-to-response associations hamper correction of covert errors (Burle et al., 2014), which increases the error rate; there is little doubt that incongruent associations will also hamper action monitoring in the more complex, uncertain, or noisy environments encountered outside the laboratory. We elaborate upon this point further in the following section.

### Basic Research Knowledge on Action Monitoring Processes May Be Useful for Applications in the Context of Working by Identifying Some of Their Limitations

The “system approach” considers that work environments may not be in accordance with the principles of organization of the operator information processing system, which, as a consequence, may increase the likelihood of errors and their undesirable outcomes (Reason, 2000, 2005; Ivensky, 2017a,b; Leveson, 2012; Bitar et al., 2018). Put differently “We design systems in which human error is inevitable and then blame the human and not the system design” (Leveson, 2012, p. 47). Changing the environment most often revealed to be much more efficient than trying to reduce human errors by manipulating rewards and punishments (Leveson, 2012, but see also Fitts, 1947 for a similar view). Therefore, even for those who are interested in application purposes (e.g., adapting machines/environment to human capabilities), there is a need for basic research knowledge regarding human information processing architecture and its limiting factors. This basic knowledge should help avoiding *a priori* situations “in which human error is inevitable.” For example, in the specific case of sharing functions between pilots and machines, Fitts, who largely concerned himself with sensorimotor activities under time pressure, already considered more than 70 years ago that “Knowledge of human abilities and limitations is also needed in deciding what equipment should be operated by the pilot and what equipment should be made entirely automatic…” (p. 30).

Obviously, the generation of errors is a well-known human limitation. Conversely, error detection, inhibition, and correction are an important ability of the human brain that can be exploited, and it must therefore be preserved. Knowledge regarding its limitations or its limiting factors may contribute to proposing ideas regarding what should be avoided if one seeks to preserve action monitoring abilities at their best level in real-world environments where sensorimotor information has to be processes under time pressure (e.g., a driver in his car, a pilot in his plane, a sprinter in his starting blocks, or a goal keeper before a penalty kick). To illustrate this point, four examples of well-established adverse conditions for action monitoring are presented in the following section. In these examples, it is reasonable to assume that real-world environments would not abolish the deleterious effect of these experimental conditions, which results can reasonably be extrapolated to real-world conditions.

#### Incongruent Stimulus-to-Response Associations and Speed-Accuracy Tradeoff

In conflict tasks, incongruent conditions generate slower RTs and increased error rates. As already indicated, a significant part of the interference effects observed on error rates is accounted for by a reduced efficiency of the supervisory system, as revealed by reduced correction ratio on incongruent conditions as compared to congruent ones. Moreover, this reduction of action monitoring efficiency on incongruent conditions is strongly amplified under speed instructions as compared to accuracy instructions (Burle et al., 2014).

#### Low Doses of Alcohol

In a conflict task, low doses of alcohol did not affect the accuracy of the subjects; interference effects were not affected either (neither on RTs nor on accuracy), and overall RTs were only slightly slower (352 ms vs. 360 ms for placebo and lowest doses, respectively, and higher doses generated larger effects). On the contrary, the effects observed on action monitoring processes were significant. The reduction of interference effects usually present after an error completely disappeared (Ridderinkhof et al., 2002); moreover these low doses of alcohol selectively reduced the amplitude of the Ne, although the error rate was not affected. The sensitivity of the Ne to low doses of alcohol contrasted with the insensitivity of short- and long-latency stimulus-evoked potentials at these same doses (Ridderinkhof et al., 2002). The effects of alcohol on the Ne were therefore specific and could not be attributed to a general depression of the electrogenesis. The strong sensitivity of these supervisory mechanisms showed that action monitoring is among the first functions upon which alcohol exerts its deleterious effects.

#### Sleep Deprivation

In a conflict task, one night of sleep deprivation weakly increased RTs, but it could be shown that this increase was completely explained by peripheral motor effects (i.e., increase of the delay separating EMG onset from the mechanical response), leaving central processes (i.e., the delay separating the occurrence of the response signal from EMG onset) unaffected. Only on incongruent associations was the error rate increased by sleep deprivation. On congruent associations, no effect on the error rate could be seen. Therefore, for congruent associations, no real effect on information processing (except on its most peripheral motor component) could be found after one night of sleep deprivation (Ramdani et al., 2013). On the contrary, the sensitivity of the supervisory system, as revealed by the amplitude of the Ne on errors and partial errors, was reduced both on congruent and incongruent associations; this effect was selective, given that other event-related components were left unaffected by this sleep deprivation. Therefore, on congruent associations, an “infraclinical” sensitivity of the supervisory system to sleep deprivation was unmasked by studying the Ne. It is not unlikely that under longer sleep deprivation or less optimal conditions than those encountered in the laboratory, this “infraclinical” sensitivity would have turned into a behavioral sensitivity.

#### Mental Fatigue

Mental fatigue (as revealed by the effects of time on task) is known to impair performance (RTs and error rates). It has been shown that this impairment also involves the supervisory system (Boksem et al., 2006); in the task used, subjects had the possibility to correct their overt errors in a 500 ms delay following their response. First, a drop in the correction rate of overt errors occurred with fatigue (39% only as compared to 73% in non-fatigue conditions). Second, PES was no longer present under fatigue conditions. Moreover, mental fatigue strongly reduced the amplitude of the Ne on errors to about one fourth of that evoked in non-fatigue conditions (see Figure 3 of Boksem et al., 2006). These results indicated that the capacities of detection and correction of the action monitoring system are dramatically reduced by mental fatigue.

#### Motivation

One might argue that, when humans work, motivation could counteract the effects of fatigue on the supervisory system. However, Boksem et al. (2006) also studied the consequences of monetary motivation on fatigue effects. Although motivation perfectly restored RTs (but left error rates unaffected), PES of fatigued but motivated subjects was modestly restored to about one third only of PES under non-fatigue conditions (see Figure 2 of Boksem et al., 2006). Regarding the Ne evoked by errors, its amplitudes was only weakly restored by motivation (see Figure 3 of Boksem et al., 2006). Therefore, motivation has an effect on the action monitoring system, but this effect, although present, is not very efficient and hardly counteracts the effects of mental fatigue.

To sum up, the effects of low doses of alcohol, mental fatigue, motivation, sleep deprivation, congruency, or speed-accuracy trade-off have been investigated in artificial but optimal laboratory conditions; they were aimed at evidencing limiting factors of the action monitoring system or unfavorable situations. One can reason that these limiting factors would also have similar (or even stronger) effects in sub-optimal situations, such as those encountered when the brain works in everyday settings. If this opinion is correct, then certain results from laboratory experiments can quite easily be extrapolated to real world situations.

### Several Laboratory Observations on Action Monitoring Still Hold True in More Ecological Tasks or Environments

As already indicated, the reported paradigms used to study information processing under time pressure are quite far from ecological tasks or environments, but can, under certain conditions, bring valuable information for application purposes. Moreover, recent efforts have been made to examine whether the results obtained so far with these paradigms could be generalized to other conditions or tasks, as certain tasks are much closer to everyday situations. We give some examples in the following section.

PES can be observed not only in simple “key press” tasks but also in more natural movements, such as in tasks requiring the reaching for and grasping of objects. This PES occurs not only after an error (Ceccarini and Castiello, 2018) but also after observing other individuals committing an error (Ceccarini and Castiello, 2019); in these cases, the grasping component of the natural movement shows a PES.

From an explicit neuroergonomic perspective, the possible influence of congruency on the Ne has also been studied for very complex visual stimuli arrays, such as a motorcycle to be detected in a complex natural image (Sawyer et al., 2017). The usual sensitivity of the Ne to correctness still holds in these complex situations. In a very natural behavior such as speech, a clear Ne is also evoked in naming tasks; the Ne is small but present on correct naming and much larger on naming errors (Riès et al., 2011, 2013). In pointing tasks *via* a cursor on a screen (very similar to some everyday tasks), a Ne can also be evoked on pointing errors; moreover, small initial pointing errors that are quickly corrected share several properties with the partial errors observed in choice RT tasks and, as expected from partial errors, they also evoke a Ne (Kieffaber et al., 2016). Even more recently—and within an environment even closer to natural situations—in a competitive-type consumer baseball video game, batting errors evoked a Ne for the batter during the 200 ms before swinging strikes, whereas this was was not the case for the pitcher. An interesting point here was that the error potentials were evoked at a moment when the ball was “flying” to the batter, that is, before the error event onset (Yokota et al., 2019). Finally, error potentials have been successfully studied not only in a simulated but also in a real-world driving task (Zhang et al., 2015).

Although very natural, all these tasks involved discrete responses, but error potentials can also be observed on errors in continuous tasks. In continuous tracking movements with a mouse controlling a cursor on a computer screen, it has been demonstrated that hand position has to be repeatedly monitored and corrected; in these continuous actions, small sub movements periodically correct the trajectory of the cursor (Pereira et al., 2017). Error potentials are evoked by these sub movements and source localization methods suggest that these error potentials are generated in the same structures as the Ne evoked by erroneous responses, such as discrete button presses (Pereira et al., 2017). Therefore, several recent data sets indicate that the action monitoring mechanisms revealed by the influence of correctness on the amplitude of error potentials in artificial laboratory tasks also hold true “…in conditions approximating those in normal daily life.” (Yokota et al., 2019, p. 11) and even hold true in a real-world driving task (Zhang et al., 2015).

These results obtained in rather ecological situations suggest that several properties of the supervisory system hold true outside of the artificial conditions of laboratory tasks. Therefore, a certain number of its abilities might be exploited in real-world situations. For example, the ability to quickly correct inappropriate behavior could be exploited to improve system safety by designing these systems in such a way that correction is always possible when humans have to process information under time pressure. This would not reduce the number of human errors but might contribute to the avoidance of some of their bad outcomes; in these cases, certain man–machine systems would become more resilient to human errors. With the development of technology in the near future, Brain Computer Interfaces could monitor error potentials in real-world situations and allow for the detection of “infraclinical” decreases of sensitivity to errors of the supervisory system of an operator before this decreased sensitivity becomes “clinical.” However, given what has been acknowledged in the section regarding the ecological validity of laboratory tasks, despite the fact that several properties of the action monitoring system hold in quite ecological conditions, real-world experiments should be conducted beforehand to verify that fast corrections or sensitivity to errors of the error potentials are still present in the complex, noisy, or uncertain environments that can be encountered when the brain works in everyday settings.

### Laboratory Physiological Measures Related to Action Monitoring May Be Used to Study the Brain at Work in Everyday Settings

Physiological indices of action monitoring processes may not only help us understand action monitoring mechanisms and their limitations, but they can also be useful for Brain Computer Interfaces (BCIs), even for those interested in the brain at work in everyday settings.

BCIs can be separated into active, reactive, and passive ones. In active BCIs, the user consciously tries to control his/her brain signals to produce appropriate input for the machine so that the machine can generate an appropriate action (e.g., turning a wheelchair to the right by imagining a movement of the right hand). Reactive BCIs take advantage of the specific brain responses to stimuli to which attention is paid; these specific responses tell the machine which stimulus the subject is paying attention to (e.g., a letter to be selected for spelling purpose). “An elegant approach to improve the accuracy of BCIs consists in a verification procedure directly based on the presence of error-related potentials…” (Ferrez and del R Millán, 2008, p. 923). In these cases, single-trial detection of error potentials tells the machine that the brain has (covertly) detected an error; this allows the machine to adapt its responses to its inputs. This aspect of the BCIs is by nature “passive” as the error potentials are not generated by the brain purposely.

In the domain of rehabilitation, several types of BCIs are developed in order to allow disabled persons to control specific devices through their brain activity only (e.g., for communication, controlling wheelchairs, controlling neuroprosthetics, etc.); usually, these BCIs are active or reactive, but adding a passive component based on the analysis of error potentials improves their efficiency (e.g., Kreilinger et al., 2012; Perrin et al., 2012; Iturrate et al., 2015).

More generally, “Passive BCIs are typically the kind of BCIs that can be used for neuroergonomics research and applications.” (Lotte and Roy, 2019, p. 3). In these BCIs, the human is not required to control explicitly any device through the brain activity of interest; this activity can nevertheless be used to improve the quality of the (active or reactive) man–machine interaction by adapting the response of the machine to the mental state of the subjects (fatigue, inattention, error detection, etc.); this occurs effortlessly for the operator who may not even be aware of it (Zander et al., 2016). Taking error potential into account in passive BCIs has proven to be extremely efficient (Ferrez and del R Millán, 2008; Chavarriaga and Millán, 2010; Chavarriaga et al., 2014; Dyson et al., 2015; Zander et al., 2016 for a review) and “… brings human cognition directly into the human-computer interaction loop, expanding the traditional notions of “interaction” (Zander et al., 2016, p. 14, 898).

With passive BCIs using error potentials, a task can even be completed by a man–machine system without the man explicitly trying to realize this task: if a subject looks at the displacement by the discrete steps of a cursor on a grid, if this subjects is informed of a final target that the cursor is supposed to reach, and if this subjects has to give a judgement on the quality of each discrete step (which is not used as input to the machine), any deviation from the optimal trajectory at each step will evoke error potentials, where the amplitude depends on the size of the deviation. By recording these error potentials and using them as input to the machine, this machine will learn to correctly guide the cursor to the target while participants are unaware of having communicated any information to the machine (Zander et al., 2016).

It is noteworthy that, on the human side of the system, the passive nature of these BCIs is an important characteristic because the additional man–machine interaction carried out by the elicitation of error potentials does not require additional processing resources; as such, this interaction, although efficient for the task, is workload free.

## Conclusion

To sum up, in sensorimotor activities developed under time pressure, the action monitoring system acts before, during, and after the action in order to take errors into account, in which “before” refers to error likelihood and need for covert inhibition of undesirable responses, “during” refers to response quality and, when necessary, the need for correction, and “after” refers to, if necessary, the need for strategic adjustments. In other words, error is taken into account by the action monitoring system at every step of response production. This indicates that the cognitive architecture, at least in sensorimotor activities, is intrinsically organized to live with a certain dose of error, whether overt or covert.

In the frame of a system approach, these facts should be taken into account for: (1) designing in the workspace, whenever possible, systems and organizations allowing the human operator to correct their own errors before they finally result in a system failure in sensorimotor activities (outside the sensorimotor domain think, for example, of the Flaujac accident); (2) identifying first which cognitive system (information processing chain, action monitoring system, or both) is affected by deleterious work situations before envisioning countermeasures against these detrimental situations; (3) identifying limiting factors of the action monitoring system to help preventing deleterious situations, whether environmental or due to “… systems in which human error is inevitable…” (Leveson, 2012, p. 47); (4) verifying in the near future to which extent certain abilities of the supervisory system identified in the artificial world of the laboratory would still hold true in real-world environments; and (5) developing, in the near future, thanks to future technical evolutions, passive BCIs, able to detect “infraclinical” drops in the efficiency of the operator’s supervisory system, either to help them in their task or to withdraw them from a potentially hazardous situation.

Taking these points into consideration may help to prevent a certain proportion or errors; it may also help to adapt systems to tolerate a certain dose of human errors in order to become more resilient to them. This would be appropriate considering, as have several other authors (e.g., Reason, 1990, 2000; Amalberti, 2001, 2013), that errors are consubstantial to the human information processing system and that “Far from being rooted in irrational or maladaptive tendencies, (these recurrent) error forms have their origins in fundamentally useful psychological processes” (Reason, 1990, p. 1).

## Author Contributions

FV, BB and TH contributed equally to the manuscript.

## Conflict of Interest

The authors declare that the research was conducted in the absence of any commercial or financial relationships that could be construed as a potential conflict of interest.

## Footnotes

^1^L’épouvantable erreur d’un seul homme.

^2^In the following all the reported results are statistically significant; for example, shorter, longer, larger, and smaller mean “significantly longer, shorter, larger, smaller, etc.”

^3^By training, subjects can establish an internal “model” of some parameters of the to-be-produced response, such as the required force, and this can be used to controlled the response force; this internal model is often called a representation. To be accurate, this model has to be updated. If it is not, the exerted force can be inappropriate.

^4^It is worth noting that not all partial correct responses unequivocally represent execution errors: this clearly depends on the experimental setup (see Questienne et al., 2018 for a convincing illustration).

^5^Note that this EEG pattern cannot be evidenced unless appropriate spatial filtering methods, such as the Laplacian transformation, are computed based on scalp potentials.

^6^Remember that the reason why children commit more errors than young adults is not related to a lower correction rate but a much higher rate of initial incorrect activations.
